# Addressing the interdependencies: the role of global health diplomacy in designing One Health governance

**DOI:** 10.1016/j.soh.2026.100161

**Published:** 2026-05-26

**Authors:** Jinkou Zhao, Alexandra Calmy, Michel Kazatchkine

**Affiliations:** a Shanghai Institute of Infectious Diseases and Biosecurity, Fudan University, Shanghai 200032, China; b Fudan Institute for Advanced Studies in Global Health, Fudan University, Shanghai 200433, China; c Institute of Global Health, University of Geneva, Geneva 1202, Switzerland; d The Graduate Institute of International and Development Studies, Geneva 1202, Switzerland

**Keywords:** Global health diplomacy, One Health governance, Pandemic preparedness, Equity and solidarity, Multilateralism

## Abstract

The convergence of human, animal and environmental health crises has exposed the limitations of fragmented governance structures. One Health faces a critical implementation gap: the persistent silos between scientific disciplines, sectors and sovereign states. This perspective argues that global health diplomacy provides the essential mechanism for designing effective One Health governance. We propose a three-part diplomatic agenda: constructing science–policy interfaces, negotiating institutional architectures, and embedding equity and solidarity as core governance principles. Without deliberate diplomatic engagement, One Health risks remaining an aspirational concept rather than an operational reality.

## Graphical abstract

**Figure:**
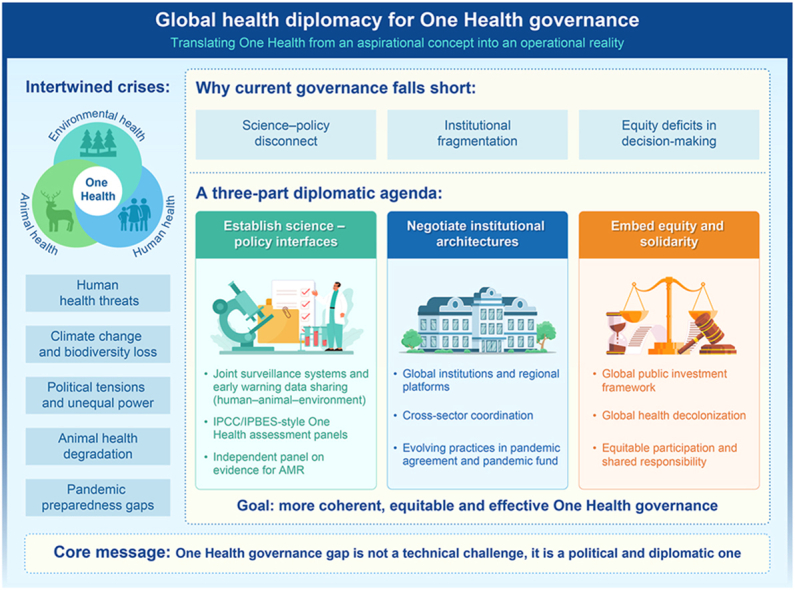

## 1 Introduction

The COVID-19 pandemic and the escalating crisis of antimicrobial resistance (AMR) have demonstrated with clarity that human, animal and environmental health are inseparable. One Health has emerged as the dominant paradigm for addressing these complex threats [1]. Yet, implementation remains profoundly challenging. One Health operates at the intersection of multiple sectors, multiple levels, and multiple actors. Each operates within its own institutional logic, legal framework, and political mandate. No single framework comprehensively integrates these domains [2].

One Health governance gap is fundamentally a political challenge, not merely a diplomatic one. Throughout this perspective, we use “diplomatic” to refer to the processes and mechanisms of negotiation, and “political” to refer to the underlying interests, power dynamics, and value conflicts that drive these processes.

Global health diplomacy provides the tools to architect the rules, norms, and institutions necessary for integrated governance [3].

The global political landscape is being reshaped by multiple forces. The United States’ (US) withdrawal from the World Health Organization (WHO) in January 2026 signals a shift from multilateralism toward selective bilateral agreements [4]. In parallel, China has expanded its bilateral health diplomacy across Asia and Africa, while Middle Eastern states have increased their engagement in global health governance. During the pandemic agreement negotiations, tensions between the European Union (EU) and the African Union (AU), distinct from US-related dynamics, revealed deep North–South divisions over benefit-sharing and technology transfer. These tensions underscore that One Health itself is not politically neutral; it was promoted strongly by Global North actors (notably the EU) while facing resistance from the African Group, reflecting broader asymmetries in global health governance [5].

**Fig. 1:**
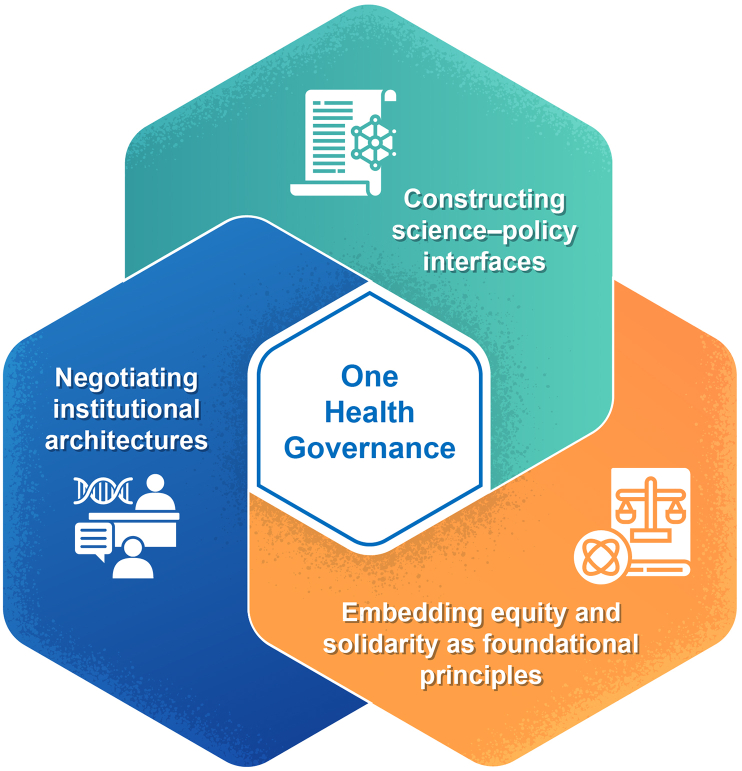
Three-dimensional diplomatic engagement in One Health governance.

In this perspective, we argue that effective One Health governance requires deliberate diplomatic engagement across three dimensions: constructing science–policy interfaces, negotiating institutional architectures, and embedding equity and solidarity as foundational principles (Fig. 1).

## 2 The challenge of One Health governance

One Health governance is defined as the “structures, processes and mechanisms that enable intersectoral coordination, collaboration and decision-making” [6]. This is linked to the definition of One Health as an “integrated, unifying approach that aims to sustainably balance and optimize the health of people, animals and ecosystems” [7].

However, three fundamental challenges impede effective One Health governance.

First, there is a science–policy disconnect. During COVID-19, early warnings from animal health surveillance were not integrated into human health preparedness [8]. The Intergovernmental Negotiating Body (INB) negotiations revealed how scientific recommendations for the Pathogen Access and Benefit-Sharing (PABS) system became entangled with sovereignty concerns and commercial interests [5,9].

Second, there is institutional fragmentation. The Quadripartite (Food and Agriculture Organization of the United Nations, United Nations Environment Programme, WHO, and World Organisation for Animal Health) collaboration represents progress, but implementing One Health at national level remains challenging. Ministries operate with distinct priorities, objectives, budgets and political accountabilities. This fragmentation has real consequences: efforts to combat AMR struggle to coordinate action across human medicine, veterinary medicine, and agriculture, with WHO noting in 2024 that only 25% of countries have fully operational multisectoral coordination mechanisms for AMR [10]. Beyond the Pandemic Fund, other financing streams, including animal health and agricultural mechanisms, remain underutilized for One Health objectives [11].

Third, there are persistent equity deficits. Wealthier countries wield disproportionate influence while developing countries questioned whether sharing pathogen data would only benefit wealthy nations and pharmaceutical corporations [12].

## 3 Constructing science–policy interfaces

The first diplomatic task is constructing effective science–policy interfaces. The Intergovernmental Platform on Biodiversity and Ecosystem Services (IPBES) offers one model, bringing scientists and policymakers together to assess evidence on biodiversity and ecosystem services. The Intergovernmental Panel on Climate Change (IPCC) similarly translates climate science for policy audiences. One Health requires comparable mechanisms that integrate human, animal, and environmental health evidence. Given the deep interconnections between climate change and One Health, a compelling question is whether the IPCC should integrate a One Health component into its core mandate, creating a more unified science–policy interface for planetary health. The Independent Panel on Evidence for Action against AMR, currently under construction, offers another promising model: its mandate to synthesize evidence across sectors could inform One Health governance design.

The Quadripartite collaboration represents a diplomatic achievement but must be complemented by mechanisms at national and regional levels. Effective One Health governance requires “whole-of-government” approaches [13].

The challenge is handling uncertainty, contested evidence, and divergent interpretations. Debates over the origins of SARS-CoV-2 illustrate this challenge precisely: inconclusive evidence creates vulnerability to politicization and manipulation. The INB and Intergovernmental Working Group (IGWG) negotiations similarly grappled with the politicization of science, particularly in debates over PABS, where scientific recommendations for transparency intersected with national sovereignty concerns and commercial interests [9,14,15].

## 4 Negotiating institutional architectures

The second diplomatic task is negotiating the institutional architectures for One Health governance. Several institutional options exist.

First, strengthen existing frameworks. The International Health Regulations (IHR) were amended in 2024, entering into force in September 2025 [16]. It is of note that the IHR revisions were politically far less contentious than the Pandemic Agreement negotiations, as they focused on technical updates to existing surveillance procedures building on decades of established state practice.

Second, create new institutional mechanisms. The Pandemic Fund, established under Indonesia's G20 presidency, represents a new financing instrument for pandemic prevention, preparedness and response [17]. Yet the question of how best to finance pandemic preparedness remains contested, with diplomatic efforts ongoing to resolve this tension.

Third, build on regional integration. Regional organizations play an increasingly important role in global health diplomacy. The AU's Africa Centres for Disease Control and Prevention, the EU's Health Emergency Preparedness and Response Authority, and regional economic communities such as Association of Southeast Asian Nations and Economic Community of West African States all provide platforms for regional One Health governance that can complement global frameworks.

Each approach requires diplomatic negotiation. Member states must agree on institutional mandates, governance structures, financing mechanisms, and accountability arrangements. They must balance national sovereignty concerns with collective action requirements. They must reconcile the interests of different ministries and stakeholders. Effective diplomacy for One Health requires connecting both issues and actors across levels, linking local surveillance data to global alert systems, bridging agricultural and health ministries within national governments, and aligning regional strategies with global frameworks. This model of connecting surveillance to objective-setting and impact measurement was successfully implemented by Joint United Nations Programme on HIV/AIDS, which integrated community-level data, national reporting, and global oversight through its Programme Coordinating Board, creating accountability mechanisms that could inform One Health governance design [3].

The pandemic agreement process demonstrated the painstaking nature of architecting global health institutions. The deferral of PABS details the IGWG underscores that institutional design is not a one-time event but an ongoing diplomatic process requiring sustained engagement and trust-building [18].

## 5 Embedding equity and solidarity in One Health governance

The third diplomatic task is embedding equity and solidarity as core principles. One Health cannot be technocratic: it must address the structural inequalities that shape health vulnerabilities and outcomes.

A growing consensus calls for “decolonizing” global health governance creating space for non-Western and indigenous perspectives. For One Health, this means recognizing that indigenous and local communities possess valuable knowledge about human–animal–environment relationships that should inform governance, not merely as beneficiaries but as co-creators of policy [19].

Equity also requires addressing power imbalances in One Health governance. While equity addresses fair distribution, solidarity adds the dimension of collective responsibility and mutual support, the recognition that in an interconnected world, no nation can be safe unless all are safe, and that shared threats require shared responses.

In practice, governance arrangements vary by institutional context. In intergovernmental settings (e.g., WHO), equal representation is standard, though not without trade-offs. However, in institutions involving significant financial contributions, equal voice is more complex due to fiduciary responsibilities. These practical constraints must be acknowledged when designing governance mechanisms [3].

The pandemic agreement negotiations brought these equity challenges into sharp focus. The core North–South divide centered on whether developing countries would share pathogen data from their territories only to see wealthy nations and pharmaceutical corporations develop and profit from vaccines without ensuring affordable access for those who shared the data. Member States failed to agree on benefit-sharing obligations and have now extended negotiations in the hope of reaching an agreement in May 2026 [14,15].

Gender equity is equally important. Women make up the vast majority of the health workforce but remain significantly underrepresented in global health diplomacy and decision-making [20]. One Health governance must ensure that women's perspectives and expertise inform policy at all levels, from local community health systems to global negotiating tables.

## 6 Recommendations: a diplomatic agenda for One Health governance

The INB experience offers practical lessons for translating the three diplomatic tasks into action. We offer the following recommendations.

For constructing science–policy interfaces, modelled on IPCC and IPBES, One Health science–policy panels may be established at national and regional levels, and at the global level, as proposed by Hobeika and colleagues for an Intergovernmental Panel for One Health [21], with formal mandates to provide integrated assessments of zoonotic risks, AMR, and environmental health threats. These panels should include diverse scientific disciplines and ensure representation from low- and middle-income countries. The Independent Panel on Evidence for Action against AMR could serve as a pilot or complementary mechanism.

Joint surveillance and data-sharing platforms should be established with governance structures that address sovereignty concerns while enabling rapid information exchange. However, the premise that such platforms can rely on universal multilateral participation must be re-examined in light of the US withdrawal from WHO in January 2026. The task for diplomatic negotiators is no longer simply to build trust, but to architect systems robust enough to survive its absence.

For negotiating institutional architectures, the financing model for One Health governance must ultimately shift from traditional donor-recipient assistance toward a Global Public Investment (GPI) framework. The High-Level Independent Panel on Financing the Global Commons articulated this vision in 2021: pandemic preparedness is a global public good that should be financed by all countries based on ability to pay, with all receiving equal benefits, a global social security model for pandemic protection. The Pandemic Fund, with nearly US$3 billion mobilized and over 85% of its portfolio now including One Health components, demonstrates that cross-sectoral coordination can be incentivized through financing design. However, its current scale remains far below the US$15 billion annual target, and its voluntary contribution model does not yet realize the GPI vision of predictable, assessed contributions from all countries. The core insight across a growing literature is that financing One Health governance cannot be charity; it must be investment in shared security, with governance reflecting equal voice and equal benefit, not donor privilege.

A potentially decisive coalition of over 500 Development Banks to fund One Health has recently been announced in One Health Summit April 2026 in Lyon, France.

Regional organizations should be strengthened as platforms for One Health governance, recognizing that regional agreements can sometimes overcome sovereignty concerns more effectively than global treaties. Regional center for disease control and prevention networks, veterinary coordination mechanisms, and environmental agreements should be explicitly linked to One Health objectives.

For embedding equity and solidarity, all One Health governance mechanisms must include decision-making structures that give low- and middle-income countries genuine voice, not merely consultation. This may require innovative governance models such as co-chairs from different income groups, rotating leadership, or weighted voting that balances gender perspectives, population and financial contributions. The diversity of perspectives on urgency and feasibility, shaped by divergent political and economic realities, must be acknowledged in governance design.

The question of legally binding vs. voluntary benefit-sharing obligations remains contested in the now extended IGWG negotiations, with legitimate concerns on both sides about sovereignty, participation, and equity. The historical record suggests that purely voluntary mechanisms have consistently failed to ensure equitable access when commercial interests and urgent public health needs collide. We therefore submit that some form of legally anchored commitment, whether treaty obligation, binding annex, or enforceable contract, is necessary to create predictability and trust, particularly for pathogen-sharing countries. The precise legal form merits continued negotiation, but the principle that benefit-sharing cannot remain purely aspirational should, in our view, be firmly established.

## 7 Conclusion

One Health governance is fundamentally a political enterprise.

The recent experience of the WHO Pandemic Agreement negotiations serves as a practical testament to both the potential and the difficulty of this diplomatic work. It shows that embedding One Health principles into binding international law is possible, but requires navigating deep geopolitical divides, reconciling competing interests, and accepting that some critical details will require continued negotiation.

The WHO Pandemic Agreement negotiation process did not resolve all tensions, particularly around benefit-sharing and technology transfer, but it demonstrated that diplomacy is the indispensable mechanism for translating the scientific reality of interdependence into political action. The recommendations we offer aim to translate these lessons into practical steps for policymakers, diplomats and health practitioners.

Perceptions of urgency differ between the Global North and South; for many low- and middle-income countries, strengthening basic health systems may be the immediate priority, with pandemic preparedness seen as a longer-term aspiration contingent on resource availability. Acknowledging this diversity is essential for equitable governance design.

Without deliberate diplomatic engagement, attentive to both political realities and practical constraints, One Health risks remaining an aspirational concept rather than an operational reality.

## CRediT authorship contribution statement

**Jinkou Zhao:** Writing – review & editing, Writing – original draft, Formal analysis, Conceptualization. **Alexandra Calmy:** Writing – review & editing, Formal analysis. **Michel Kazatchkine:** Writing – review & editing, Conceptualization.

## Funding

None.

## Declaration of competing interest

Authors declare we do not have any conflicts of interests.
